# Advanced functional nanofibers: strategies to improve performance and expand functions

**DOI:** 10.1007/s12200-022-00051-2

**Published:** 2022-12-19

**Authors:** Xinyu Chen, Honghao Cao, Yue He, Qili Zhou, Zhangcheng Li, Wen Wang, Yu He, Guangming Tao, Chong Hou

**Affiliations:** 1 School of Optical and Electronic Information, Huazhong University of Science and Technology, Wuhan, 430074 China; 2 Wuhan National Laboratory for Optoelectronics, Huazhong University of Science and Technology, Wuhan, 430074 China; 3 State Key Laboratory of Materials Processing and Die and Mould Technology, School of Materials Science and Engineering, Huazhong University of Science and Technology, Wuhan, 430074 China; 4 Research Institute of Huazhong University of Science and Technology in Shenzhen, Shenzhen, 518063 China; 5 Department of Electrical Engineering and Computer Science, Massachusetts Institute of Technology, Cambridge, 02139 USA

**Keywords:** Functional nanofiber, Nanofiber fabrication, Nanofiber structure, Nanofiber materials, Nanofiber assembly

## Abstract

Nanofibers have a wide range of applications in many fields such as energy generation and storage, environmental sensing and treatment, biomedical and health, thanks to their large specific surface area, excellent flexibility, and superior mechanical properties. With the expansion of application fields and the upgrade of application requirements, there is an inevitable trend of improving the performance and functions of nanofibers. Over the past few decades, numerous studies have demonstrated how nanofibers can be adapted to more complex needs through modifications of their structures, materials, and assembly. Thus, it is necessary to systematically review the field of nanofibers in which new ideas and technologies are emerging. Here we summarize the recent advanced strategies to improve the performances and expand the functions of nanofibers. We first introduce the common methods of preparing nanofibers, then summarize the advances in the field of nanofibers, especially up-to-date strategies for further enhancing their functionalities. We classify these strategies into three categories: design of nanofiber structures, tuning of nanofiber materials, and improvement of nanofibers assemblies. Finally, the optimization methods, materials, application areas, and fabrication methods are summarized, and existing challenges and future research directions are discussed. We hope this review can provide useful guidance for subsequent related work.

**Graphical abstract:**

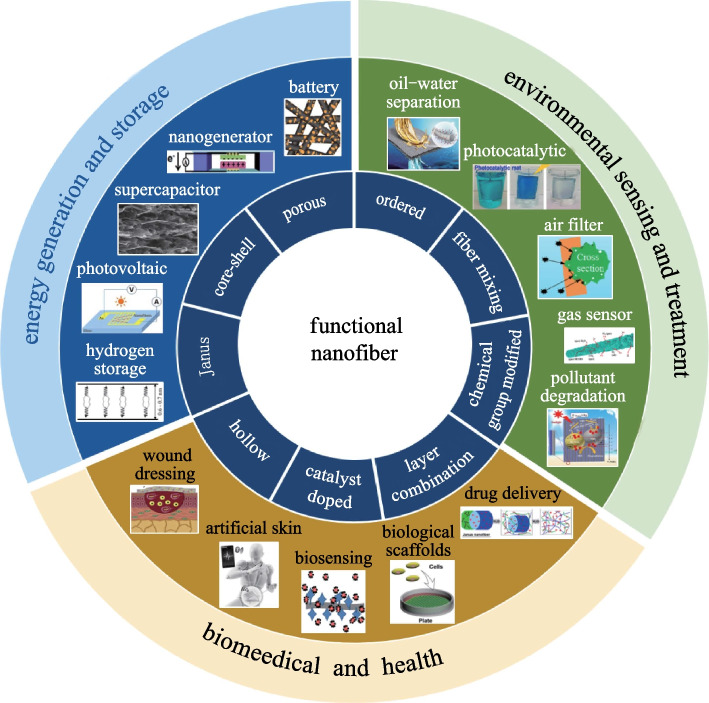

## Introduction

Nanofiber, in a broad sense, refers to ultrafine fibers with a diameter of less than 1000 nm. Benefitting from the nano-size, nanofiber has the characteristics of a large specific area, large aspect ratio, small size effect, superior mechanical properties, and is widely used in many fields, including air filter, drug delivery, and battery [1–3]. For one thing, owing to the one-dimensional (1D) morphology and ultrafine size, nanofibers have properties that neither bulk materials nor films have. For example, the specific surface area of nanofibers is 1−2 orders of magnitude larger than that of films [4], which gives them unique advantages in the fields of gas sensing, supercapacitors, photocatalysis, etc. [2, 5–9]. For another, novel electronic, optical, and catalytic properties appear as the size of the material is reduced to the nanometer scale. For example, Wang et al. [10] found that a single indium phosphide nanowire exhibited highly polarized photoluminescence and photodetection. This opens up extensive research on the interaction of light with nanofibers [11–14]. Last but not least, nanofibers have good flexibility and toughness. Research revealed that spider silk is composed of multiple nanofibers, with the breaking force of every single one estimated to be ≈ 120 nN. And when 2500 nanofibers composed of spider silk were put together, their toughness is five times higher than that of steel wire [15]. Nanofiber especially played a critical role during the COVID-19 pandemic as it is the key material of non-woven textile used to make masks that prevent the virus from entering the human respiratory system. Pores between nanofibers can effectively block viruses while ensuring breathability, so they are widely used in air filters, artificial skin, wound dressing, etc. [1, 16, 17].

**Fig. 1:**
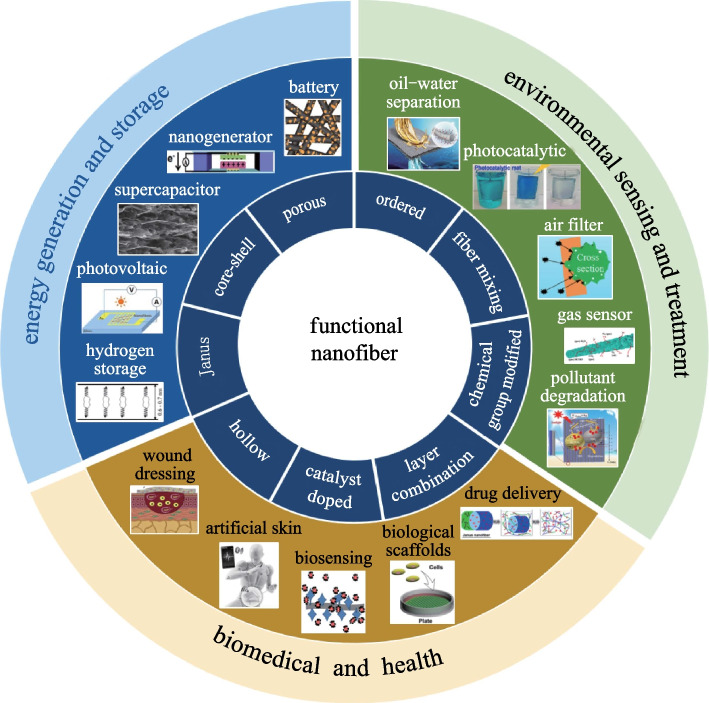
Wide range of applications of nanofibers

Research on nanofibers has boomed in recent decades and a large variety of novel fiber synthesis approaches have been developed (electrospinning [18–23], thermal drawing [24–26], melt blowing [27–29], sea island [30–32], chemical route [33–36], direct drawing [37, 38], etc.) to explore incorporation of new materials (polymer [39], metal [40], ceramic [41], semiconductor [25], etc.) for diverse functionalities. Applications include but are not limited to air filters, gas sensors, supercapacitors, photocatalysts, etc. [1, 8, 39, 42, 43], as shown in Fig. 1. In this review, a broad range of works on the preparation and application of nanofibers are reviewed. First, the preparation methods of nanofiber are introduced. Then the strategies to improve the properties of nanofibers are summarized, including design of nanofiber structures, tuning of nanofiber materials, and improvement of nanofiber assemblies. Finally, the challenges and the outlook of nanofibers are discussed with the purpose of peeking into the future of this promising field.

## Fabrication of nanofibers

**Fig. 2:**
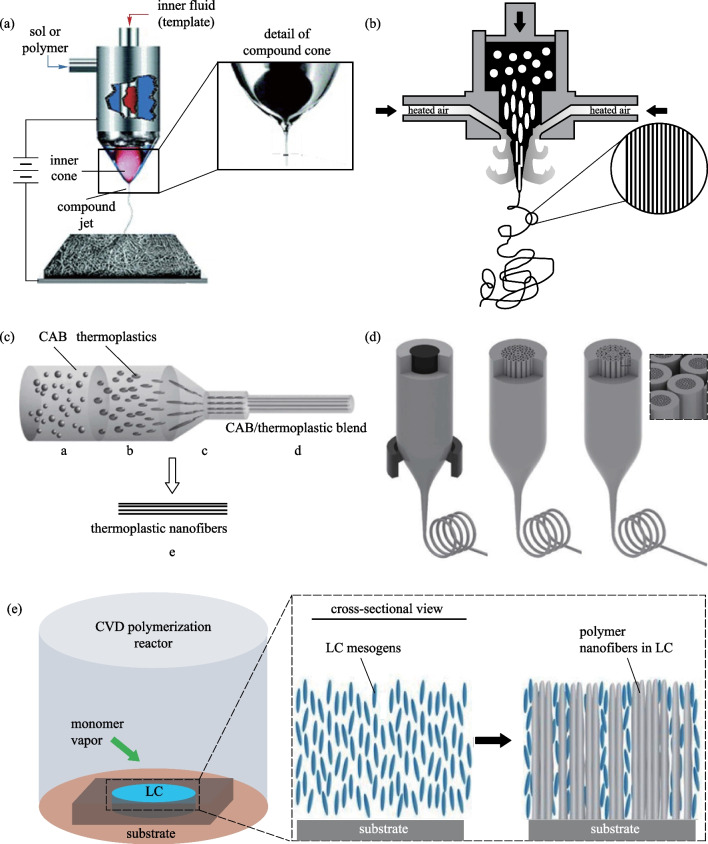
Schematic diagram of common nanofiber preparation methods. **a** Electrospinning [55]. **b** Melt blowing [27]. **c** Sea island [49]. **d** Thermal drawing [25]. **e** Chemical synthesis [33]

As mentioned before, a lot of approaches have been used to prepare nanofibers. Among those, electrospinning is one of the most common methods, which can produce continuous nanofibers [44–46]. The principle of electrospinning is that a strong electric field induces the charged jet of the fluid to eject from the tip of a Taylor cone, the solvent evaporates and leaves the fibers [47], as shown in Fig. 2a. Through adjustment of concentration and process parameters, electrospinning can produce fibers with diameters ranging from tens of nanometers to dozens of microns. Electrospinning can prepare nanofibers of various materials including polymer, metallic oxide, and semiconductors. It can also prepare porous [1], core–shell [16], Janus [48], and other structural nanofibers by structural design. Generally speaking, electrospinning is suitable for materials that can be dissolved in solvents.

Melt blowing is another commonly used method, mostly applied to prepare nonwoven fibers [27]. In this method, the polymer is first heated to a high temperature to melt, then the molten polymer is blown by compressed gas into fibers [28], as shown in Fig. 2b. The resulting fiber size is mostly affected by the air-to-polymer mass flow rate and is also affected by other factors such as material selection, orifice diameters, processing conditions, and die geometries [29]. The advantages of melt blowing are its high efficiency, low cost, and mass production. Normally, the diameter of melt-blown nanofibers is not uniform, and the fiber structure is difficult to be diversified. Currently, melt blowing is only suitable for thermoplastic polymers [31].

An interesting method of preparing nanofibers is the sea-island method [31]. This method can be combined with the melt blowing, as shown in Fig. 2c [27]. It utilized immiscible polymer blends of target polymer and another sacrificial polymer. In Fig. 2c, the target polymer is poly [ethylene-*co*-(glycidyl methacrylate)] (PE-*co*-GMA), and the sacrificial polymer is cellulose acetate butyrate (CAB) [49]. The target polymer was blended in the sacrificial polymer matrix as micro-sized spherical dispersions, and the well-dispersed micro-ellipsoids were subsequently extruded through a spinneret die and elongated into nanosized fibers. After removal of the sacrificial polymer, the target nanofibers were obtained in a form of continuous yarns. The sea-island method has the advantages of simplicity and efficiency, and the nanofibers produced using this method are aligned in a regular manner, which is important for application that needs good mechanical properties [50], or oriented growth of cells and tissues [51], etc. At present, sea-island method is suitable for a small number of material combinations, and the resulting fiber has a short length and uneven diameter distribution [32].

The thermal drawing method is used to draw a thick preform into a fine fiber in a furnace, which is often used to prepare communication fibers. In recent years, some groups have used the thermal drawing method to prepare nanofibers. In 2008, Deng et al. [26] found that sulfide film would crack into nanofibers during the thermal drawing process. In 2011, Yaman et al. [25] reported a new iterative co-drawing process, which can prepare globally oriented, endlessly parallel, axially and radially uniform semiconducting and piezoelectric nanowire or nanotube arrays hundreds of meters long, with nanowire/nanotube diameters less than 15 nm, as shown in Fig. 2d. In 2011, Kaufman et al. [24] used the iterative co-drawing process to prepare nanofibers with a diameter of only 5 nm. Thermal drawing is an effective method that can prepare globally oriented and ultra-long nanofibers in large quantities. To prepare nanofibers by the thermal drawing method, the glass transition temperature and viscosity of the core and cladding material combination need to be matched [52].

Chemical methods, including chemical oxidative polymerization [34], chemical vapor polymerization [33], plasma-induced synthesis [53], etc., can prepare nanofibers of various materials, such as polymers [54], metal oxides [53], liquid crystals [33], and other materials, which have been used in applications such as optical sensors, chemical sensors, biosensors. [34]. Generally, the length of the nanofiber prepared by the chemical method is on the micrometer scale, and it is difficult to carry out structural design for nanofibers during the chemical process. The schematic diagram of nanofiber synthesis via chemical vapor polymerization is shown in Fig. 2e.

In addition to the above commonly used preparation methods, many special methods are also used to prepare nanofibers, including direct drawing [37], carbon dioxide laser supersonic drawing [56], in situ deposition [57], centrifugal jet spinning [58], self-assembled [59], each with its own advantages and disadvantages. For example, Hasegawa and Mikumi [56] improved the mechanical properties of polymer nanofibers by means of carbon dioxide (CO_2_) laser supersonic drawing. Behrens et al. [57] used solution blow spinning to generate conformal nanofiber mats/meshes on any surface in situ. In actual applications, it is necessary to select a suitable preparation method according to practical requirements.

## Design of nanofiber structures

### Porous nanofibers

**Fig. 3:**
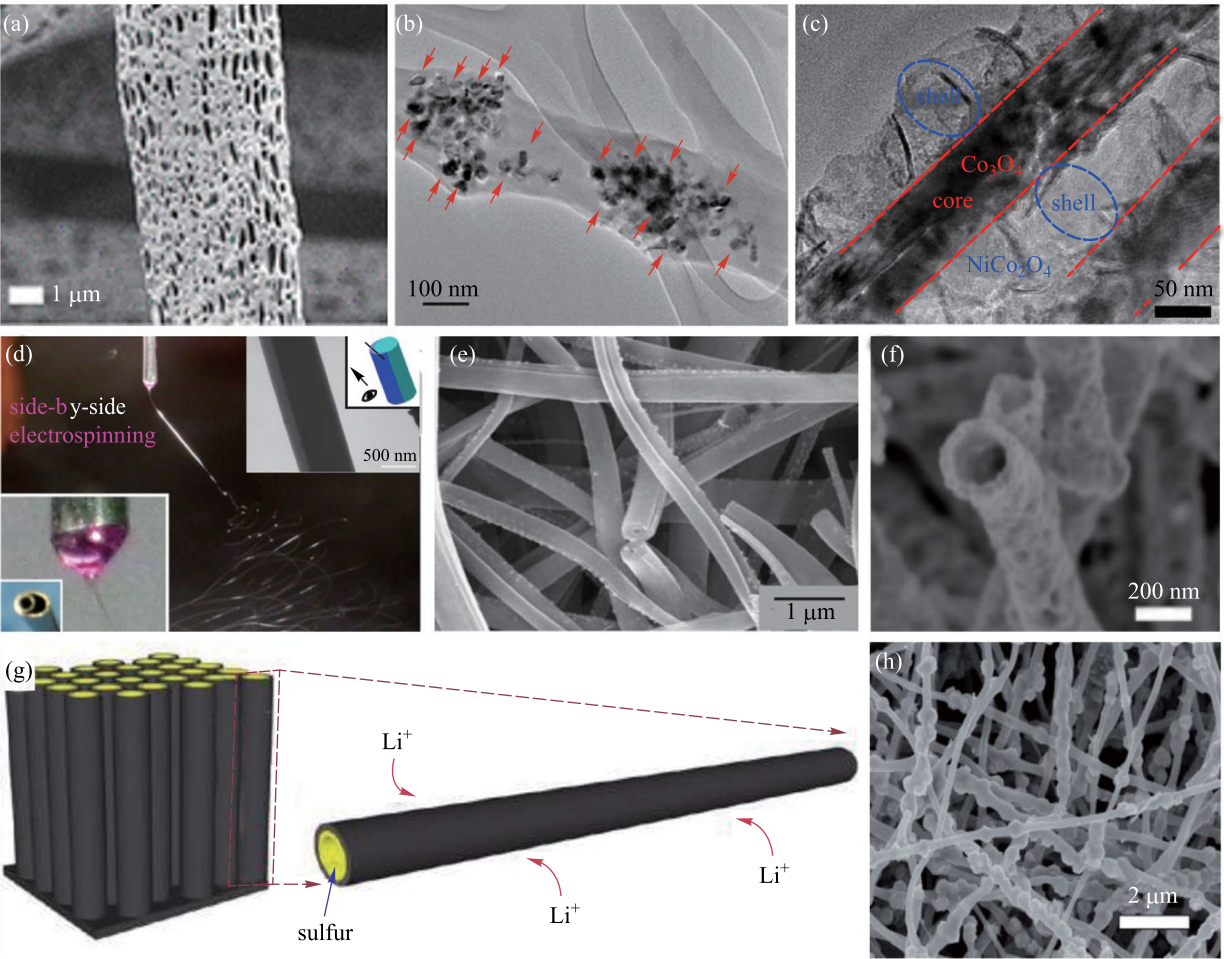
Various structures of nanofibers. **a** SEM photo of porous nanofibers [60]. **b** TiO_2_ photocatalysts are anchored on the porous structure for water purification [64]. **c** Core–shell nanofibers prepared by hydrothermal method [68]. **d** Two needles are combined and the SEM image of the prepared fiber [69]. **e** Electrospun Janus nanofibers for which one side is carbon nanofiber embedded with CoO_*x*_ nanoparticles and the other side is smooth carbon nanofiber [48]. **f** SEM photo of hollow nanofibers [70]. **g** Schematic diagram of S coated hollow carbon nanofiber [71]. **h** SEM photo of necklace-like nanofiber [72]

One of the most important advantages of nanofibers is the large specific surface area. Preparation of porous structure on the surface or inside of nanofibers can further increase their specific surface area, as shown in Fig. 3a. There are many ways to prepare porous nanofibers; the two most commonly used methods are sacrificial polymer [1] and sacrificial solvent [60]. In the method of sacrificial polymer, two polymers (sometimes a polymer and a salt ion [61]) are dissolved in the same solvent and nanofibers are prepared by electrospinning or other methods. After that, the sacrificial polymer is dissolved and the porous nanofiber is obtained. The principle of sacrificial solvent is similar to that of sacrificial polymer, except that the latter approach has two solvents, one is regarded as solvent-L (with lower boiling point and can dissolve the polymer) and the other is regarded as solvent-H (with higher boiling point and cannot dissolve the polymer). During the spinning process, solvent-L evaporates quickly and solvent-H remains in the nanofiber. Then a heating process removes solvent-H to obtain nanofibers that have pores throughout them [60].

Porous nanofibers show good performance in the fields of filtration [1, 60], adsorption [61], supercapacitors [62, 63], and catalysis [64] because of their large specific surface area. In 2020, Xie et al. [1] used electrospinning to prepare PI nanofibers with special wrinkled porous structures and a high specific surface area of 304.77 m^2^/g was achieved. The scattering of particulate pollution on the wrinkled surface can effectively improve the filtration efficiency. The wrinkled porous PI nanofiber filter displayed a high PM_0.3_ removal efficiency of 99.99% with a low pressure drop of 43.35 Pa at room temperature. In 2011, Ji et al. [62] synthesized porous carbon nanofibers (CNFs) with encapsulated sulfur (S) via electrospinning, carbonization, and solution-based chemical reaction–deposition. When the porous nanofibers were used as cathodes in rechargeable Li/S cells, it maintained a stable discharge capacity of about 1400 mAh/g at 0.05 C. The excellent electrochemical performance was attributed to the high electrical conductivity and the extremely high surface area. In 2018, Lee et al. [64] prepared titanium dioxide doped porous poly (vinylidene fluoride) (PVDF) nanofibers, as shown in Fig. 3b, and applied them for the degradation of organic pollutants in water. The hydrophobic nature of PVDF allows nonpolar organic contaminants to concentrate on its surface where TiO_2_ photocatalysts are anchored.

### Core–shell nanofiber

Core–shell structure is a commonly used structure of functional nanofibers [65]. There are two main methods to prepare core–shell nanofiber; one is coaxial electrospinning (CO-ES) [16, 43, 66] and the other is thermal growth [6, 7, 67, 68]. Coaxial electrospinning is derived from electrospinning, and a key component in the process is the coaxial needle: during electrospinning, one solution enters a core tube and the other enters a cladding tube, and then the core–shell nanofiber is obtained by electrospinning. It is worth noting that the coaxial needle can be used not only in electrospinning, but also in other spinning methods, such as melt blowing and solution blow spinning [17]. In the thermal growth method, a nanofiber is first fabricated and then soaked in a solution to induce the formation of cladding, as shown in Fig. 3c. This method is also sometimes called hydrothermal method.

In core–shell nanofibers, the core and shell can implement different functions individually or work together to achieve one goal. In 2018, Lin et al. [43] used coaxial electrospinning to prepare a PVDF-HFP (Polyvinylidene Fluoride-hexafluoropropylene)@PDMS (Polydimethylsiloxane) ion gel core–shell nanofiber. In this fiber, the shell is the protective layer to prevent evaporation of water from the hydrogel in the core, and the hydrogel in the core can realize piezoelectric sensing. In 2018, Yue et al. [6] used electrospinning and hydrothermal method to prepare Mo_2_C@SrTiO_3_ core–shell nanofiber. Since the bandgap of semiconductor Mo_2_C and semiconductor SrTiO_3_ is different, this core–shell structure forms a heterojunction and has excellent photocatalytic performance for water splitting. Similarly, Wan et al. [7] prepared hierarchical In_2_O_3_@SnO_2_ core–shell heterojunction nanofiber for high-efficiency formaldehyde detection.

### Janus nanofiber

Janus nanofiber is a single nanofiber composed of two materials. A common method of preparing Janus nanofiber is side-by-side electrospinning, as shown in Fig. 3d. Two types of matching electrospinning precursor solutions are assembled with two electrospinning spinnerets and Janus nanofiber with biphasic components is thereby prepared [69].

In Janus nanofibers, the coordination of two fiber materials enables complex functionalities. In 2020, Cao et al. [48] electrospun Janus type CoO_*x*_//C nanofibers with one side of the nanofiber being carbon nanofiber embedded with CoO_*x*_ nanoparticles and the other side being smooth carbon nanofiber, as shown in Fig. 3e. The fabricated Janus nanofiber can be employed as an electrocatalyst for the oxygen reduction reaction. In 2020, Sun et al. [73] electrospun [TiO_2_/C]//[Bi_2_WO_6_/C] carbon-based Janus nanofiber. In this nanofiber, TiO_2_ and Bi_2_WO_6_ form heterojunction that can absorb both ultraviolet light and visible light for hydrogen production and photodegradation. In 2018, Wang et al. [69] mixed polyvinylpyrrolidone K10—sodium dodecyl sulfate (an unspinnable fluid) and polyvinylpyrrolidone K90—helicid (an electrospinnable fluid) to co-electrospin and create a Janus nanofiber with water-insoluble drug loaded inside, and achieved a rapid drug release system based on the Janus nanofiber. In 2020, Yang et al. [74] prepared PVP-CIP//EC-AgNPs (polyvinylpyrrolidone-ciprofloxacin//ethyl cellulose-silver nanoparticles) Janus nanofiber, where CIP is an antibiotic that ensures a strong antibacterial effect at the initial stages of wound healing and AgNPs prevents further bacterial infections of the wound.

### Hollow nanofiber

Hollow nanofibers have a hollow tubular structure, as shown in Fig. 3f. There are many ways to prepare hollow nanofibers, and one of the most important methods is coaxial electrospinning. First, the core–shell nanofiber is produced by coaxial electrospinning, and the core material will evaporate during electrospinning, such as N,N-Dimethylformamide (DMF) [55], or during pyrolysis in the subsequent heating process, such as PVP [75]. It is worth mentioning that electrospinning can not only prepare single hollow nanofibers but also prepare multi-channel hollow nanofibers [76]. Other methods, such as thermal drawing [25] and template method [71], can also prepare hollow nanofibers.

Hollow nanofibers with inner and outer surfaces provide larger specific surface area and can provide more active sites for chemical reactions, which has advantages in sensors [70, 77], electrodes [8, 71], catalysts [39, 75, 76] and so on. In 2017, Liu et al. [77] prepared Ce_0.6_Mn_0.3_Fe_0.1_O_2_ layered porous hollow nanofibers as efficient anodes for mixed direct carbon fuel cells. The hollow structure provides more opportunity for CO permeability and effectively improves catalytic activity during CO oxidation. In 2019, Hwang et al. [70] used hollow nanofibers for gas sensing. In addition, a p–n junction between Cu/CuO and ZnO was realized on the nanofiber shell, which improved the sensitivity of the nanofiber to CO gas at 300 °C. The inner surface also protects the adsorbed material from dissolving. In 2011, Zheng et al. [71] prepared sulfur-coated hollow carbon nanofibers for a rechargeable lithium battery with high specific capacity by using a template method. The hollow structure can effectively capture sulfur in the inner wall of carbon nanofibers and significantly improve the reversible capacity, as shown in Fig. 3g.

Besides, benefiting from the structural features including plentiful active sites, continuous conducting pathways, and benign mass transfer channels, hollow nanofibers are suitable for electrocatalysis [75]. In 2019, Wang et al. [39] prepared N-doped hollow carbon nanofibers (HCNFs) that exhibited remarkable catalytic degradation of tetracycline in the peroxymonosulfate (PMS) activation system. In 2019, Gao et al. [75] prepared N,P-co-doped hollow carbon nanofibers (N,P-HCNFs) that exhibited an excellent tri-functional electrocatalytic activity for the oxygen reduction reaction, the oxygen evolution reaction, and the hydrogen evolution reaction. In 2020, Zhu et al. [76] prepared multi-channel V-doped CoP hollow nanofibers to enlarge the exposure of active sites, to facilitate the electron transfer, and to tune the electronic structure of the active sites, resulting in an enhancement of the hydrogen evolution reaction.

Apart from the structures discussed above, there are other nanofiber structures, including multifaceted, necklace-like, and multi-walled nanofibers [44]. In 2019, Kong et al. [72] synthesized a necklace-like N-doped carbon-wrapped mesoporous silicon nanofiber (NL-Si@C) network by electrospinning and magnesiothermic reaction, as shown in Fig. 3h. The necklace-like structure effectively avoids particle agglomeration and structural collapse. The polymer shell can be carbonized during the subsequent conversion process, providing conductive channels for electron transport. Nanofibers with this structure can be used as anodes for lithium-ion batteries.

## Tuning of nanofiber materials

### Polymer blended nanofiber

**Fig. 4:**
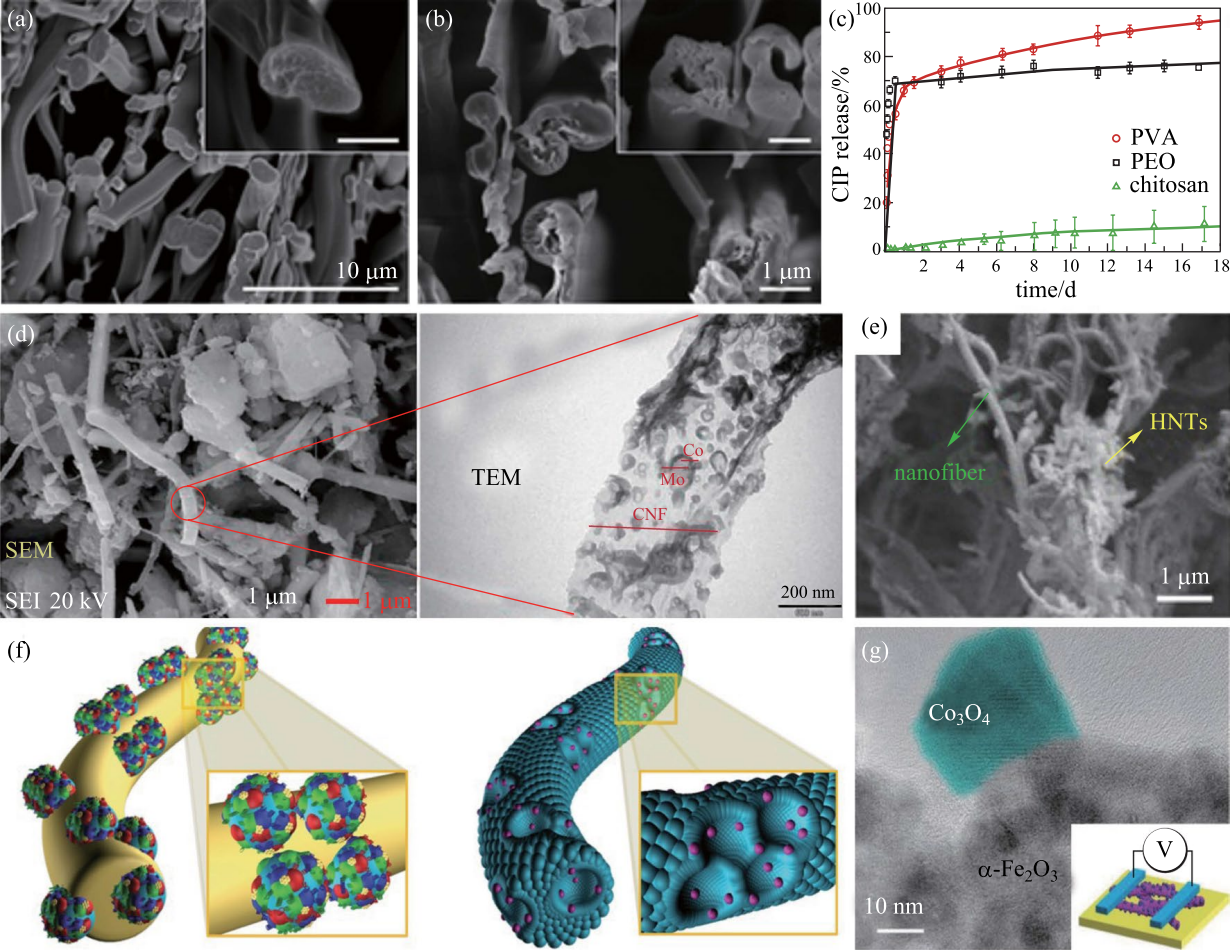
**a**, **b** SEM images of nanofiber cross sections before **a** and after drug release for 18 days **b**. **c** Release profiles of CIP from blended nanofibers with 10 wt % of PVA, PEO, or chitosan added to PMMA [81]. **d** SEM image and TEM image of AlCNF and MoCo catalyst on the nanofiber [86]. **e** SEM image of HNTs on PI nanofiber [87]. **f** Schematic illustrations of as-spun composite nanofiber comprising W precursor, PVP, and catalyst-decorated PS colloids (the left picture) and catalytic nanopartical-loaded porous WO_3_ nanofibers after calcination (the right picture) [88]. **g** TEM image of hetero-nanofiber with Co_3_O_4_ and α-Fe_2_O_3_ crystal phases. Inset: schematic of a device for electrical measurements [89]

Polymer-blended nanofibers belong to multi-component systems in which two or more materials are fused to form new nanofibers [78, 79]. Polymer-blended nanofibers are fabricated to achieve multifunctionality and enhance the properties of nanofibers [80]. For example, by selecting suitable materials, the mechanical properties of nanofibers can be improved. Moreover, the degradation rate of nanofibers can be regulated by adjusting the ratio of materials. These properties are important for wound dressings and drug delivery. In 2016, Zupančič et al. [81] mixed polymethyl methacrylate (PMMA) with different hydrophilic polymers. Different mixing ratios can achieve different ciprofloxacin release rates. Nanofiber before and after drug release for 18 days is shown in Fig. 4a, b. A comparison of release profiles for different polymers is shown in Fig. 4c. In 2017, Yao et al. [82] mixed soft, elastic, slowly degrading polycaprolactone (PCL) with stiff and rapidly degrading polylactic acid (PLA), so that the advantages of both polymers could be retained, which can be used as scaffold of osteogenic differentiation of human mesenchymal stem cells (hMSCs) and cranial bone formation. In 2018, Lobo et al. [83] incorporated poly (ethylene glycol) (PEG) and gelatin methacryloyl (GelMA) into PCL nanofibers, and significantly improved the mechanical properties and hydrophilic properties of PCL nanofibers. This nanofiber can be used for a variety of orthopedic applications. In 2022, Sun et al. [84] prepared polylactic acid/polyethylene glycol (PLA/PEG) micro/nanofiber fabric by a post-drafting melt blown process. The PEG can reduce the *T*_g_ (glass transition temperature) of PLA, improve the mobility of PLA molecular chains, reduce the complex viscosity of PLA/PEG blends, and play a role in plasticization. This improves the tensile strength of the fiber.

### Catalyst doped nanofiber

Catalysts play an important role in the oxygen reduction reaction [85], pollutant treatment [9, 65, 86], energy conversion [73] and other fields. Improvement of catalytic efficiency has, for a long time, been the focus of scientific research. One of the effective means to improve catalytic efficiency is to increase the specific surface area of the catalyst while avoiding its agglomeration. In this case, distributing the catalyst on the surface of the nanofibers has huge advantages. As the carrier of the catalyst, nanofibers can effectively avoid the agglomeration of the catalyst, and at the same time the large specific surface area can give full play to the performance of the catalyst. In 2018, Al-Hammadi et al. [86] prepared a novel alumina doped carbon nanofiber (AlCNF) and distributed MoCo catalyst on the fiber surface for the hydrodesulfurization (HDS) reaction of dibenzothiophene (DBT), as shown in Fig. 4d. The enlarged specific surface area significantly improved catalytic efficiency. In 2019, Xu et al. [87] utilized short electrospun polyacrylonitrile (PAN) and polyimide (PI) nanofibers as a backbone to support halloysite nanotubes (HNTs), making them have good mechanical elasticity and stability. It effectively prevented agglomeration of the catalyst and enhanced its dispersivity, as shown in Fig. 4e. The structure can be used as a novel dye adsorbent and catalyst carrier.

In some cases, nanofibers not only provide support for catalysts but also have other functions, such as providing conducting pathways, and direct participation in chemical reactions. In 2016, Panomsuwan et al. [85] prepared nitrogen-doped nanoparticles-carbon nanofibers (NCNP-CNFs) as cathode catalysts for oxygen reduction reaction (ORR) in fuel cells. In this structure, NCNPs with meso/macroporosity provide active sites for ORR, while CNFs serve as a highly conductive pathway for charge transport. In 2016, Choi et al. [88] employed polystyrene (PS) colloid-template to discretize catalysts, which were then attached to WO_3_ nanofibers by high-temperature heat treatment, as shown in Fig. 4f. This catalyst-modified WO_3_ nanofibers exhibit excellent sensing performance for H_2_S and acetone, with potential applications for portable diagnosis of halitosis and diabetes. In 2018, Wang et al. [89] prepared Co_3_O_4_ nanoparticles with specific (112) crystal facet in α-Fe_2_O_3_ nanofibers, and the so-formed heterostructured nanofibers can be used as high-performance flexible sensing devices of ammonia gas. In this structure, the (112) crystal facet in the Co_3_O_4_ nanoparticles produces a large number of highly active sites, which are favorable for the selective adsorption of ammonia molecules, thereby improving the sensitivity, as shown in Fig. 4g.

### Chemically modified nanofiber

Chemical modification improves the performance and function of nanofibers by reactions, such as polymerization, grafting, oxidation, and hydrolysis, or other methods to obtain specific functional groups on the surface of the nanofibers. Compared with ordinary physical adhesion and doping, the connection between substances through chemical bonds is more reliable, and the composite material prepared therefrom is more durable.

**Fig. 5:**
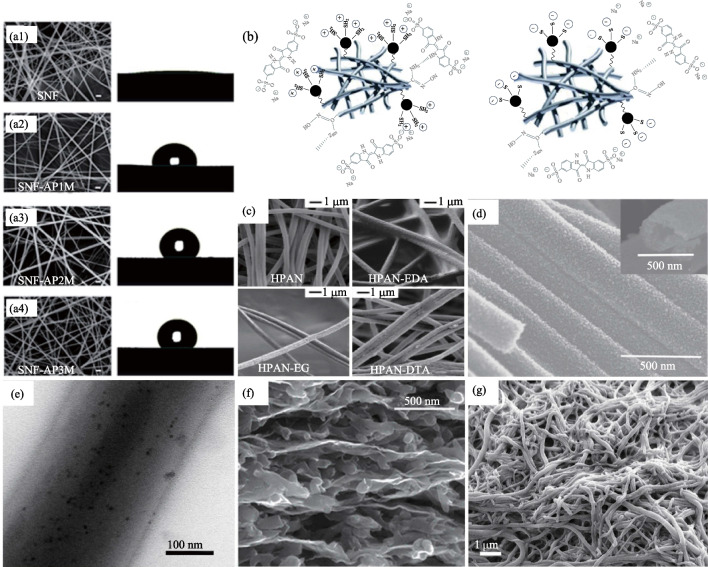
**a** SEM images and contact angles of SNF modified by different APTS concentrations [92]. **b** Schematic representation of adsorption (left) and desorption (right). Black particles are representative of Fe_3_O_4_ nanoparticles [94]. **c** SEM images of electrospun hydrolyzed PAN nanofibers (HPAN) and chemically modification with EDA, EG, or DTA [95]. **d** Well-arranged nanofiber arrays of La-doped SnO_2_ nanofibers [103]. **e** TEM image of a 280-nm-diameter QD/PS nanofiber [97]. **f** Cross-section SEM image of CCG mixed PANI-nanofibers [2]. **g **SEM image of PA6 filaments coated with PVA-co-PE nanofiber [49]

In the chemical modification of nanofibers, a significant requirement is to change the hydrophilic or hydrophobic properties of their surfaces to realize their applications in water treatment [90], medicine delivery [91], and tissue engineering [92]. In 2019, Wang et al. [90] prepared the super-hydrophilic polyphenylsulfone nanofiber membranes where plasma treatment oxidized the polymer on the surface of nanofibers, endowing the membranes with excellent hydrophilicity with a contact angle of 0°, providing an application for water treatment. Such high hydrophilicity plays a crucial role in enhancing the water flux through the membrane. In 2020, Cheng et al. [91] prepared sponges with Janus character using cellulose nanofibers that exhibit different wettability characteristics at different facets. Epoxy groups were added to the surface of cellulose nanofibers modified by γ-glycidyloxypropyl-trimethoxysilane, which improved the hydrophilicity. Vinyl groups were added to the surface of vinyl-trimethoxy-silane modified cellulose nanofibers, which improved the hydrophobicity. The hydrophilic layer absorbs water from the blood to accelerate blood clotting, and the hydrophobic layer prevents blood penetration into the tissue and exerts pressure on the wound. Chen et al. [92] employed (3-aminopropyl) trimethoxysilane (APTS) to amino-functionalized silica nanofibers (SNFs). After modification, the SNFs can significantly change the surface from hydrophilic to hydrophobic due to the presence of a large number of amino groups on the surface, as shown in Fig. 5a. Therefore, neural stem cells cultured on modified SNFs substrate showed a higher degree of proliferation than those cultured on unmodified SNFs substrate.

Based on the high porosity and high specific surface area, chemically modified nanofiber can be used to purify water and adsorb harmful substances by changing the functional groups on the surface. It has been previously shown that the adsorption of metals ions and organic dyes can be achieved by using compounds containing functional groups such as amidoxime, carboxyl, amino, phosphoric, and imidazoline, which have a complexing effect on metal ions and dyes [93]. In 2018, Yazdi et al. [94] reported that the surface of amidoximated polyacrilonitrile (APAN) could be coated with Fe_3_O_4_ nanoparticles modified by 3-mercaptopropionic acid (Fe_3_O_4_-MPA) through chemical cross-linking. The modified APAN/Fe_3_O_4_-MPA nanofibers resulted in a maximum loading capacity of 154.5 mg/g for the indigo carmine (IC) dye. The adsorption efficiency of indigo carmine was kept almost constant when the nanofibers were reused several times, as shown in Fig. 5b. In 2018, Morillo Martín et al. [95] used polyacrylonitrile (PAN) as the base polymer of electrospinning and prepared nanofibers with the ability to selectively remove heavy metal ions from sewage. Three ion-selective nanofiber materials were prepared by two steps of grafting polymerization, which included hydrolysis and chemically modification with ethylenediamine (EDA), ethyleneglycol (EG), or diethylenetriamine (DTA), as shown in Fig. 5c. In 2015, Zhao et al. [96] prepared phosphorylated polyacrylonitrile nanofibers by grafted modification and electrospinning. The phosphate-based grafted PAN nanofibers could adsorb Cu^2+^, Pb^2+^, Cd^2+^, Ag^+^, and other metal ions in an aqueous solution.

### Other composite material based nanofiber

In addition to the polymer blends and catalyst-doped nanofibers described above, many other materials have been incorporated into nanofibers, thereby optical [97], electrical [2], sensing [98], and other properties of dopants can be embedded into nanofibers [99, 100]. In this case, the nanofibers often provide protection and support, while avoiding particle aggregation. In 2019, Zhu et al. [101] incorporated TiO_2_ into polyvinylidene fluoride (PVDF) nanofibers, and the doping of TiO_2_ particles promoted ordered arrangement of moleculars and increased β polymorphism, which significantly improved piezoelectric performance of the nanofibers in geophone applications. In 2018, Cao et al. [102] synthesized La-doped SnO_2_ nanofibers as gas sensors, and when the Sn/La molar ratio was 100:7, the sensitivity was increased by nearly 10 times. On this basis, they further improved the ordering of the nanofibers, thereby enhancing the sensing performance, as shown in Fig. 5d [103]. In 2011, Meng et al. [97] mixed CdSe/ZnS quantum dots with polystyrene (PS), as shown in Fig. 5e, to prepare nanofibers as humidity sensors with fast response and low power consumption. In 2010, Wu et al. [2] mixed chemically converted graphene (CCG) and polyaniline nanofibers (PANI-NFs) to make supercapacitors with high capacitance and high cycling stability, as shown in Fig. 5f. In 2020, Chen et al. [49] prepared natural melanin/poly (vinyl alcohol-*co*-ethylene) (PVA-co-PE) nanofibers/PA6 composite fibers as twisted and coiled fiber-based actuators, as shown in Fig. 5g. In this case, the entangled PVA-co-PE nanofibers enhanced the mechanical properties of the composite fibers. In 2022, Ma et al. [98] combined flexible polyacrylonitrile nanofibers (PANF), functional polyvinyl alcohol (PVA) polymer, conductive carbon nanotubes (CNTs), and hydrophobic octadecylamine functionalized reduced graphene oxide (ODA-rGO) to form layered porous aerogels, achieving the fusion of self-cleaning, oil–water separation, and piezoresistive sensing. In 2022, Yao et al. [104] obtained the ZnO@nanofiber hybrid air filter by coating the mixture of flower-like ZnO superstructures and the PVA-co-PE nanofiber suspension on the surface of meltblown polypropylene (PP) nonwoven, with the electret charge eliminated. The flower-like ZnO superstructures significantly reduced the pressure drop of the pure PVA-co-PE nanofiber air filter and improved filtration efficiency.

## Nanofiber assembly

### Orderly aligned nanofibers

**Fig. 6:**
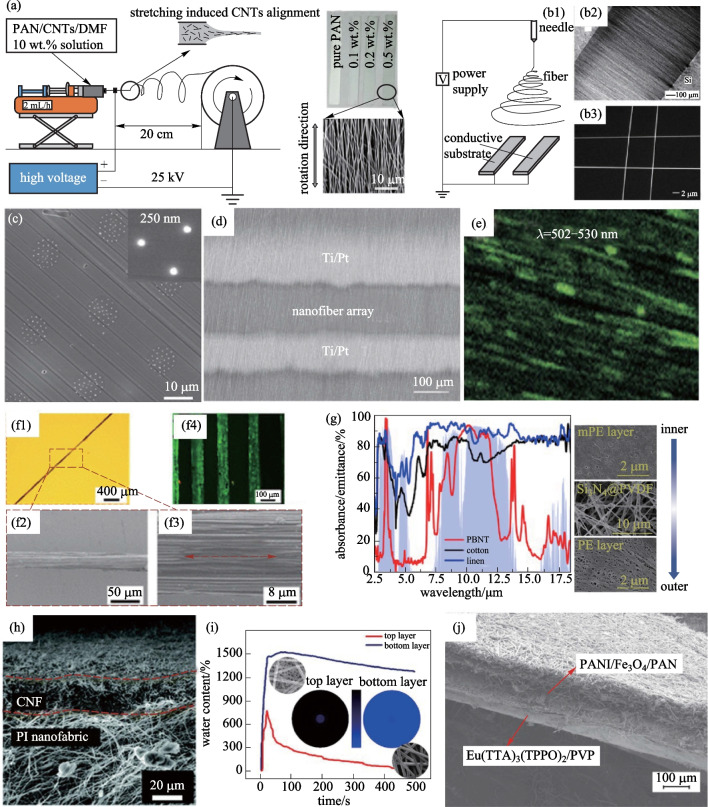
**a** Electrospinning collection by a drum collector: experimental set-up and the obtained aligned PAN/CNTs nanofibers [115]. **b** (1) Schematic illustration of the set-up that used an electric field to align nanofiber arrays. The collector was composed of two conductive substrates separated by a gap; (2) Dark-field optical micrograph of PVP nanofibers collected across the gap formed between two silicon strips; (3) SEM image of a 2 × 2 array of fibers [108]. **c** SEM image of the cross-section of indefinitely long uniform nanofiber arrays prepared by the hot drawing method [25]. **d** SEM images of NTO nanofiber array sensor between Ti/Pt electrode [114]. **e** Scanning probe fluorescence image shows quite a homogeneous distribution of green-emitting molecules [12]. **f** (1), (2), (3) are aligned NFYs and (4) is a fluorescent merged image of aligned and elongated C2C12 cells fully attached to the aligned NFYs core within the 3D hydrogel shell [51]. **g** Microstructure of the PBNT from the inner to outer surfaces and IR absorbance/emittance curves of the PBNT, cotton, and linen. The light blue area refers to the atmospheric transmittance [116]. **h** Cross-section of the CNF@PI Janus battery separator [117]. **i** Moisture tester performance of commercial nonwoven polyethylene terephthalate/PA-Ag composite membranes with 60 μm nanofiber/net thickness [118]. **j** Cross-section SEM image of the dual-layered membrane [119]

Many methods have been developed to achieve ordered nanofibers, some use a drum yarn collector [105, 115], as shown in Fig. 6a, or a disk yarn collector, or adding an external electric field [108], as shown in Fig. 6b. In 2004, Fennessey and Farris [106] used a high-speed (8.1–9.8 m/s) rotating drum collector to obtain unidirectionally aligned PAN nanofibers with superior mechanical properties. The ultimate strength and modulus of the twisted yarns reached a maximum of 162 ± 8.5 MPa and 5.9 ± 0.3 GPa, respectively. In 2020, a plant-inspired soft bistable structure was developed by Lunni et al. [107], and a rotating drum was used to produce aligned polyethylene oxide (PEO) nanofibers with anisotropic mechanical properties, whose Young’s modulus measured in the parallel direction was markedly higher than that in the orthogonal direction.

Some process the nanofiber after the electrospinning using the stretching method to get orderly nanofiber. In 2004, Li et al. [41, 108] generated large-area uniaxially aligned nanofibers by collecting them over a gap formed between two conductive silicon substrates. The charged electrospun poly (vinyl pyrrolidone) (PVP) nanofibers driven by electrostatic interactions were stretched to form a parallel array across the gap. Similar to stretching method, heat drawing method can also prepare ordered nanofibers. Yaman et al. [25] used multiple iterations of thermal drawing to achieve globally oriented, axially and radially uniform arrays of semiconductor and piezoelectric nanofibers. The fibers can be hundreds of meters long and less than 15 nm in diameter, as shown in Fig. 6c. The “flowing water bath” is also a common method to collect ordered nanofibers. Liu et al. [109] prepared continuous bundles of aligned PAN nanofibers with smooth surfaces, reduced diameters (by 56%), and improved crystallinity (by 72%) through a flowing water bath collector at 97 °C. Templating is another method for nanofiber alignment. The introduction of nanofillers as templates into the nanofiber precursors is generally an effective approach. Ma et al. [111] obtained continuous bundles consisting of aligned PAN copolymer nanofibers by using a flowing water system and subsequently stretching. Compared to the conventional microfibers (made of the same PAN copolymer), the prepared nanofibers were ~ 20 times thinner in diameter; and had a smoother surface, higher density, and higher degrees of crystallinity and macromolecular orientation. The stretched bundle of aligned PAN copolymer nanofibers might be an innovative type of precursor for making continuous carbon nanofiber bundles with superior mechanical properties (particularly the strength).

Mechanical properties are key parameters that should be considered for practical application of nanofibers. Previous research revealed that structural characteristics (size, distribution, etc.) largely determine the mechanical strength of nanofibers. Generally speaking, orderly arranged nanofibers exhibit significantly different mechanical properties compared to randomly arranged ones. In 2011, Arshad et al. [50] reported that the tensile strength of randomly arranged carbon nanofibers dramatically reduced at high temperature due to fiber rupture at early stage induced by stress mismatch with the surrounding amorphous carbon. Therefore, it is an effective way to enhance mechanical properties by producing orderly arranged nanofibers. In 2019, Kim et al. [112] investigated structural perfection of PAN based nanofibers and pointed out that aligned fibers displayed larger continuous domains, more compact structure, and fewer defects than the control sample (randomly arranged), and as a result, superior strength. In 2018, Lee et al. [110] revealed that PAN nanofibers showed a unique tendency toward the lying-down configuration on the CNT surface, which indicated the possibility of linear alignment and large-scale PAN-PAN assembly.

Nanofibers can generate and detect polarized light when their size is comparable to or smaller than the wavelength of light. In 2001, Wang et al. [10] found that a single indium phosphide nanowire exhibited highly polarized photoluminescence and photodetection. Taking advantage of this feature, people have attempted to use nanofiber as display materials which exhibited low energy consumption and high brightness. In 2020, Liao et al. [14] reported electrospun polarized light emission from non-conjugated polymer fibers that were highly aligned with nearly perfect uniaxial orientation. The nanofiber array showed polarized deep blue luminescence with a photoluminescence quantum yield of about 31% and an anisotropy of photoluminescence of 0.37 when it was exposed to 340 nm wavelength ultraviolet light. In 2011, Yin et al. [13] reported the preparation of a uniaxially aligned poly[2-methoxy-5-(20-ethylhexyloxy)-1,4-phenylene vinylene] (MEH-PPV) nanofiber array based on electrospinningx process, with a polarized red-emission spectrum, as shown in Fig. 6e. In 2018, Chakrabarty et al. [11] reported that co-assembled anisotropic CdSe–CdS nanowires and the self-assembled fluorescent nanofibers (2,3-didecyloxyanthracene) formed organogels that exhibited sharply polarized red luminescence (exposed to green light). Owing to its anisotropy, the nanofibers can be used for probing optical images of nano-objects or surfaces.

Benefiting from high orderliness, nanofibers have special applications in oriented growth of cells and tissues, and conductive pathways as well. Biocompatible nanofibers with aligned structural characteristics show advantages in assisting the oriented growth of cells and tissues. In 2015, Wang et al. [51] presented 3D cellular alignment and elongation of C2C12 myoblasts in a core–shell column. The sheet composite scaffolds were built by encapsulating a piece or layers of aligned nanofiber yarns (NFYs) cores within a hydrogel shell cured through photocrosslinking, which performed very well in applications related to skeletal muscle regeneration, as shown in Fig. 6f. Aligned carbon nanofibers also present promising applications in electrochemical energy generators and storage devices due to their excellent conductivities, extremely large surface areas, and structural stability [113].

The ordered nanofiber array is beneficial for electron transport, which offers improved device performance over random nanofibers in terms of sensing response and time for recovery. In 2017, Li et al. [114] used a modified electrospinning technique, followed by hot-press and calcination to prepare Ni-doped SnO_2_ (NTO) nanofiber array for NO_2_ detection, as shown in Fig. 6d. It showed a high response (resistance ratio is 90.3 under 20 ppm), fast response and recovery (40 and 18 s), and excellent gas selectivity. In 2017, Nikfarjam et al. [5] fabricated a single aligned nanofiber of pure TiO_2_ and gold nanoparticle (GNP)-TiO_2_ for CO detection, and the CO concentration thresholds for the pure TiO_2_ and GNP-TiO_2_ nanofiber were about 5 ppb and 700 ppt, respectively.

### Nanofiber mixing and layers combination

Mixing different nanofibers or combining different nanofiber layers can achieve more complex functions, often achieving the effect of 1 + 1 > 2, making them widely used in energy, environmental purification, and biomedical applications. In 2019, Kong et al. [117] used a combination of carbon nanofiber (CNF) layer and polyimide (PI) nanofiber layer as a battery separator, as shown in Fig. 6h. The CNF layer facing sulfide cathode served for blocking/converting polysulfides. The PI nanofiber layer facing Li anode with a highly porous structure had good wettability with electrolyte, which can promote the transport of Li^+^. This combination achieved a high initial capacity (1393 mAh/g at 0.1 A/g) and a coulombic efficiency of 99.6%. In 2016, Oh et al. [120] prepared a Janus-faced battery separator to improve the high-temperature cycle performance of the rapid charge/discharge reaction. Thiol-functionalized SiO_2_ microsphere cushion wrapped by polyvinylpyrrolidone/polyacrylonitrile nanofibers served as a support layer to capture heavy metal ions dissolved in liquid electrolyte. A thin mat of polyetherimide nanofibers wrapped by nanotubes served as the top layer to improve the kinetics of redox reactions.

The combination of different functional fibers provides the possibility for smart fabrics. In 2020, Song et al. [116] prepared a polymer-based nanophotonic textile (PBNT) using radiative cooling, which possesses high spectral selectivity (*η* = 5.12), IR absorbance/emittance (*ε*_AW_ = 87.31%) and sunlight reflectance (*ρ* = 93.28%), as shown in Fig. 6g. In 2020, Ahmed Babar et al. [118] prepared composite membranes by the rational combination of commercial nonwoven polyethylene terephthalate as hydrophobic layer, and polyamide and silver nanoparticles (PA-Ag) composite nanofiber/nets as a hydrophilic layer via a one-step electrospinning process for directional moisture transport, as shown in Fig. 6i. In 2016, Wang et al. [119] prepared a double-layer film based on polyaniline (PANI)-Fe_3_O_4_ nanoparticles (NPs)-polyacrylonitrile (PAN) electrical-magnetic bifunctional nanofibrous layer and Eu(TTA)_3_(TPPO)_2_-polyvinylpyrrolidone (PVP) photoluminescent layer, as shown in Fig. 6j. They thus integrated multiple functions to realize the regulation of conductivity, magnetism and photoluminescence. In 2022, Liang et al. [121] prepared composite papers with Janus structure based on MOF-derived CoNi@C-silver nanowires/cellulose nanofibers (MAg/CNF) by a combination of two-step vacuum filtration and hot pressing. The composite papers exhibited distinct electrical differences on both sides, resulting in the electromagnetic Interference (EMI) shielding effectiveness (SE) reaching 82 dB in the X-band.

Nanofiber plays an increasingly important role in modern society [122, 123]. The state-of-the-art design strategies to improve their performance and functions have been comprehensively reviewed in this article. Different preparation methods, modified methods, results and applications are given in Table 1.

**Table 1:** Different preparation methods, modified methods, results and applications

Preparation method	Modified method	Result	Application	Refs.
Electrospinning	Mixing with sacrificial polymer	PI, TiO_2_@PVDF, CNF-S porous nanofiber; NiCo_2_O_4_ and carbon, CMF, N, P/carbon, V/CoP hollow nanofiber	Filter, adsorption, degradation of pollutants, electrode, supercapacitor, anode, electrocatalysts	[1, 8, 62, 64, 75–77]
	Mixing with sacrificial solvent	PLC porous nanofiber	Filter	[60]
	coaxial electrospinning	PLLA/PGS, PDMS-iongel/PVDF-HFP core–shell nanofiber	Wound dressing, sensor and nanogenerator	[16, 43]
	Electrospinning and hydrothermal growth	In_2_O_3_@SnO_2_, CoS_2_-C@MoS_2_ core–shell nanofiber	Gas sensor, electrocatalyst	[7, 67]
	Electrospinning and dipcoating	SrTiO_3_@Mo_2_C core–shell nanofiber	Photocatalyst	[6]
	Side-by-side electrospinning	CoOx/C, PVP, PVP-CIP//EC-AgNPs, PVP/Zein Janus nanofiber	Electrocatalyst, drug delivery, wound dressing	[3, 48, 69, 74]
	Subsequent pyrolysis	ZIF-8/PAN, Cu/CuO@ZnO hollow nanofiber	Catalysts	[39, 70]
Template method	Using porous silicon films as the template	glassy porous CNF	Adsorption and detection,	[124]
	Using liquid crystals films as the template	Polymer nanofiber	Having properity like wettability, intrinsic photoluminescence, biodegradability, and surface charge, etc	[33]
	Using anodic aluminum oxide (AAO) membranes as the template	S/carbon hollow nanofiber	cathode	[71]
Chemical synthesis	self-assembly	p-6P/6T ordered nanofiber	Photonic sensor	[12]
	Plasma-induced technique	CuO nanofiber	Nanomaterials synthesis	[53]
	amino groups grafted	NH_2_-ASEP nanofiber	water treatment, membrane separation, catalysis, pH responsive delivery	[125]
Thermal drawing	Capillary instability,	Se, As_2_Se_3_ nanofiber	Photo detector	[26]
	iterative co-drawing	As-Se/PVDF core–shell, Ge-As-Se-Te, Se, As_2_Se_3_ nanofiber; PVDF nanotubes	Photo detector, flexible nanowire sensors, nanowire-based phase-change memory, reconfigurable field-effect transistors	[24, 25]
Melt blowing	Polymer blending	PBT, PECTFE nanofiber	filter	[27]
Sea-island method		PE-co-GMA, thermoplastic polymer nanofiber	Actuator	[49]
Solution plasma process	Nitrogen-doped carbon nanoparticle	NCNP-CNF	Cathode	[85]
direct drawing	Quantum-Dot-Doped	CdSe-ZnS QD doped PS nanofiber	Optical sensor	[97]

## Challenges and prospective

So far, many materials have been integrated into nanofibers, and the applications of nanofibers have covered many aspects of life and production. Although a lot of original studies have been carried out, there are still issues that call for better solutions.

1. For large-scale fiber preparation, current technologies are not mature enough and their robustness needs to be improved. For example, in the field of energy generation and storage, although nanofibers with high efficiency and high-density energy storage have been achieved in the laboratory stage, there are still many problems to be solved for mass production.
2. It is desirable to achieve more complex and diverse functions. For example, in the field of environmental sensing and governance, how to realize the combination of filtration, sensing, catalysis, reusability and other functions deserves further exploration.
3. At present, smart wearable devices are the future trend. Realizing the combination of nanofibers and electronic devices for detection, early warning, diagnosis and treatment of human health is one of the promising development directions.
4. Realizing the compatibility of nanofiber production methods with traditional industries is one of the major challenges. For example, in the textile industry, nanofibers are one to two orders of magnitude smaller than conventional textile fibers in diameter and are easy to break and difficult to handle. Current methods for fusion of different nanofibers are mainly mixing and multi-layer stacking. These two methods cannot fully utilize the advantages of different nanofibers. If the regular arrangement or orderly weaving of multiple functional nanofibers can be effectively realized, it will be a huge breakthrough in the application of nanofibers.
5. It is important to identify “killer” applications of nanofibers, which proves the core value of this technology. The emerging applications of nanofibers have been summarized in many fields, but most of them are at the early proof-of-concept stage, and there is still a long way to go before commercialization.

Nanofibers are the frontier of nanotechnology. As technology advances, all of these challenges will be overcome, and we will likely see stronger, more functional nanofibers used more widely in human life.
